# Pulmonary Mucormycosis: An Emerging Infection

**DOI:** 10.1155/2012/120809

**Published:** 2012-12-12

**Authors:** Mohammed Muqeetadnan, Ambreen Rahman, Syed Amer, Salman Nusrat, Syed Hassan, Syed Hashmi

**Affiliations:** ^1^Department of Internal Medicine, University of Oklahoma Health Sciences Center, Oklahoma City, OK 73104, USA; ^2^Division of Infectious Diseases, Henry Ford Hospital, Detroit, MI 48202, USA

## Abstract

Mucormycosis is a rare, but emerging, life-threatening, rapidly progressive, angioinvasive fungal infection that usually occurs in immunocompromised patients. We present a case of pulmonary mucormycosis in a diabetic patient who was on chronic steroid therapy for ulcerative colitis. Early recognition of this diagnosis, along with aggressive management, is critical to effective therapy and patient survival. The delay in diagnosis of this rapidly progressive infection can result in mortality.

## 1. Introduction

Mucormycosis is caused by the ubiquitous saprophytic fungi of the order Mucorales and class Zygomycetes. The most common organisms causing mucormycosis belong to the genera *Rhizopus*, *Lichtheimia*, and *Mucor* [1]. It is associated with high mortality and debilitating morbidity. Though uncommon, its incidence appears to have increased in recent years. We present a case of mucormycosis as the cause of nonresolving pneumonia in a diabetic patient, who was on a chronic steroid therapy for his ulcerative colitis. With the increasing incidence of this potentially fatal condition it is pertinent that physicians maintain a high index of suspicion especially in the immunocompromised. 

## 2. Case Report

A 68-year-old man with a past medical history significant for ulcerative colitis, diabetes mellitus, and a recent hospitalization for pneumonia represented seven days after discharge with cough, chest pain, and fevers. He was on chronic steroid therapy for ulcerative colitis. He had completed a ten-day course of cefdinir and azithromycin. However, he never fully recovered and reported progression of his symptoms after discharge. His temperature at home was 102.5 F. He reported severe dyspnea on exertion, a persistent dry cough, and right-sided, pleuritic chest pain.

Physical exam revealed a temperature of 100.9°F, an oxygen saturation of 95% on 3 L nasal cannula, and decreased breath sounds, crackles, dullness to percussion, and egophony at the right lung base. 

Labs were significant for white blood cell count of 16,000 with 90% neutrophils and 8% bands. Chest radiograph showed right lower lobe consolidation. He was empirically started on vancomycin and piperacillin/tazobactam. Blood cultures were negative. Bronchoscopy revealed a soft tissue mass obstructing the bronchus intermedius suggestive of malignancy or fungal pneumonia. Biopsy demonstrated abundant fibrinopurulent exudates and ulcerated bronchial wall with ischemic necrosis. The admixed were numerous nonseptate hyphae, suggestive of mucormycosis. The patient underwent pneumonectomy and was started on amphotericin B and caspofungin, but he returned 2 months after discharge with further exacerbation of his symptoms. Chest CT showed spread of infection to the left upper lobe. His hospital course was complicated by amphotericin B related cholestasis and renal failure. Mucormycosis spread to the pericardium and care was withdrawn. Unfortunately, he succumbed to the infection within 5 months of diagnosis.

## 3. Discussion

Nonresolving pneumonias are a relatively common clinical problem, but they can present a challenge to the managing physician. Alternative pathogens, such as a fungal infection, need to be considered when antibiotic regimens targeting traditional bacterial etiologies fail to achieve a cure. 

Numerous predisposing factors have been suggested for mucormycosis. They include ketoacidosis and uncontrolled diabetes mellitus [2], renal failure [3], solid tumors [4, 5], acquired or congenital neutropenia [6], immunosuppressive therapy [7], and solid organ transplantation [8]. Healthcare-associated mucormycosis [9] has also been reported in relation to ostomy bags, adhesive bandages, and wooden tongue depressors. In our patient, diabetes mellitus and chronic steroid use were the predisposing factors. 

The most common presentation is rhino-orbital-cerebral involvement [10], followed by pulmonary infection. The other anatomic forms [11] of this disease include gastrointestinal, cutaneous, renal, and disseminated mucormycosis. The type of presentation usually depends on the underlying host conditions.

Pulmonary mucormycosis occurs after inhalation of fungal sporangiospores [11]. Mucormycosis agents being angioinvasive cause infarction of the affected tissues [12]. Fungus causes necrosis and can invade tissue to spread locally or disseminate systemically. It can present with mild to severe symptoms such as fever, cough, chest pain, dyspnea, hypoxia, and hemoptysis. Pulmonary mucormycosis has a predilection to invade the adjacent organs such as the pericardium, chest wall, and mediastinum. Invasion of the large mediastinal vessels can lead to massive hemoptysis, which could occasionally be fatal. 

Diagnosis can be particularly challenging in part because of its relative rarity. On chest imaging, pulmonary mucormycosis may present with focal consolidation, lung masses, pleural effusions, or multiple nodules [13]. Direct histological examination of the tissue biopsy remains the gold standard for diagnosis. The histopathological findings reveal irregular broad nonseptate hyphae and spores. 

Effective management requires a 3-pronged combination of medical and surgical modalities along with correction of the predisposing underlying condition(s). Amphotericin B or its newer lipid formulation—liposomal Amphotericin—B (L-AmB) along with extensive surgical debridement to remove the necrotic tissue, remains the mainstay of therapy [14]. Despite aggressive treatment, invasive mucormycosis carries a high mortality rate. The overall mortality in those with pulmonary mucormycosis is high (76%) [15]. Thus it is important that clinicians maintain a high degree of suspicion for pulmonary mucormycosis in case of immunocompromised patients with nonresolving pneumonia. Early diagnosis and aggressive treatment might reduce the mortality associated with this devastating fungal infection.
